# Anxiety, Psychological Resilience, and Physical Activity Among University Students: Mediation and Latent Profile Analyses

**DOI:** 10.3390/healthcare14091246

**Published:** 2026-05-05

**Authors:** Hui Peng, Syed Ghufran Hadier, Yuze Zhang, Syed Danish Hamdani, Syed Muhammad Zeeshan Haider Hamdani

**Affiliations:** 1 Tianjin Sport University, 301617, Tianjin, China; penghui@tjus.edu.cn; 2 School of Physical Education and Big Health, Sichuan University of Business and Technology, Chengdu 611745, China; 3 School of Physical Education, Shanxi University, Taiyuan 030006, China; zhangyuze@sxu.edu.cn; 4 Health Sciences and Sport, University of Stirling, Stirling FK9 4LA, UK; 5 Department of Sports Sciences, Bahauddin Zakariya University, Multan 60800, Pakistan; zeeshanhamdani@bzu.edu.pk

**Keywords:** anxiety, psychological resilience, physical activity, university students, Pakistan

## Abstract

**Objective:** This study examined whether psychological resilience mediates the association between anxiety and PA among Pakistani university students and, as a secondary aim, explored heterogeneity in these relationships using person-centred approach. **Methods:** A cross-sectional survey was conducted among 770 university students (aged 18–26 years) from six cities in Punjab, Pakistan, between May and December 2025. Participants completed validated measures of anxiety (GAD-7), psychological resilience (CD-RISC-10), and self-reported PA. Correlation, regression, mediation, and latent profile analyses were performed, controlling for gender and academic year. **Results:** Anxiety was significantly and negatively correlated with both resilience (r = −0.475, *p* < 0.001) and PA (r = −0.378, *p* < 0.001), while resilience was positively associated with PA (r = 0.419, *p* < 0.001). Physical activity was significantly predicted by anxiety (B = −0.383, *p* < 0.001) and resilience (B = 0.174, *p* < 0.001) in regression models, explaining 21.7% of the variance in PA. The mediation analysis revealed that resilience explained 39% of the total effect of anxiety on PA, indicating partial mediation. LPA revealed three distinct profiles: an Anxious–Inactive group (27.1%) characterised by high anxiety and low resilience; a Moderately Distressed–Moderately Active group (47.0%) with intermediate levels; and a Resilient–Active group (25.8%) with low anxiety and high resilience. **Conclusions:** Lower physical activity among Pakistani university students is partly explained by reduced psychological resilience in the context of anxiety. These findings highlight the importance of resilience as a protective resource and support integrated mental health and physical activity interventions in resource-limited university settings.

## 1. Introduction

University life marks a critical and turbulent transition in young adulthood. The shift from school to higher education brings sustained academic pressure, financial uncertainty, and complex social demands [1], conditions that collectively elevate psychological distress and increase vulnerability to anxiety [2,3]. Anxiety is now among the most prevalent mental health concerns in university populations globally, with longitudinal evidence suggesting that symptom severity rises more sharply during this period than in non-student peers of the same age [2,4]. What makes this particularly concerning from a public health perspective is not anxiety in isolation, but its downstream effect on health behaviour [1,5]: students experiencing higher anxiety tend to engage less in physical activity (PA), even though PA is well-established as protective for both physical and mental health [6,7].

Despite consistent evidence of this inverse association, the psychological mechanisms that explain why anxiety translates into behavioural disengagement remain insufficiently understood, particularly in non-Western contexts where structural and cultural factors compound these dynamics [8] One underexplored mechanism is psychological resilience. Defined as the capacity to regulate emotions, adapt to adversity, and sustain goal-directed behaviour under psychological stress [9], resilience has been identified as a mediating variable in related behavioural pathways: it has been shown to explain the relationship between physical exercise and depression in Chinese university students [10] and to mediate the link between mental health literacy and anxiety [11]. However, whether resilience functions specifically as a mechanism through which anxiety disrupts PA engagement and whether this pattern holds in non-Western university populations, remains a critical gap in the literature [12].

This gap is especially significant in the Pakistani context. Pakistani university students face not only universal academic stressors but also contextual pressures including intense institutional competition, limited access to mental health services, financial insecurity, and sociocultural stigma surrounding psychological distress [13]. In a lower-middle-income setting with constrained mental health infrastructure, anxiety is likely to go undetected and unaddressed [14], and prevalence estimates suggest anxiety may be higher in Pakistani universities than the global average [15]. Critically, where formal psychological support is scarce, personal psychological resources such as resilience may become the primary determinant of whether a student sustains health-promoting behaviours or withdraws from them [16]. This contextual reality makes the study of resilience not merely theoretically interesting but practically urgent.

Beyond individual-level associations, existing research has relied predominantly on variable-centred methods that examine average relationships across the full sample, obscuring the possibility that distinct subgroups of students experience quite different combinations of anxiety, resilience, and PA. A person-centred approach such as latent profile analysis (LPA) offers a complementary lens: rather than estimating average effects, it identifies naturally occurring subgroups whose profiles of these three constructs differ in meaningful ways [17]. To our knowledge, no study has simultaneously tested the resilience mediation pathway and applied LPA to characterise heterogeneous student profiles within this framework, particularly in a South Asian university population.

The present study was therefore designed to address these gaps. The primary aim was to examine whether psychological resilience is consistent with a mediating role in the association between anxiety and PA among Pakistani university students. The secondary aim was to identify latent subgroups based on joint patterns of anxiety, resilience, and PA, and to examine how these profiles differ. Three hypotheses guided the study: (H1) that higher anxiety would be associated with lower PA; (H2) that the data would be consistent with psychological resilience as a partial mediator of this association, such that anxiety is linked to diminished resilience, which in turn is associated with reduced PA; and (H3) that distinct latent profiles would emerge, differing meaningfully in their configurations of anxiety, resilience, and PA.

### 1.1. Theoretical Framework

This study is grounded in Conservation of Resources (COR) theory, which provides a unified framework for understanding how psychological stress disrupts health behaviour. COR theory proposes that individuals are motivated to acquire, protect, and replenish valued resources whether cognitive, emotional, or social and that stress arises when these resources are threatened or lost [18]. Within this framework, anxiety functions as a chronic resource-depleting stressor: by sustaining heightened threat appraisal and emotional reactivity, it progressively erodes the cognitive and motivational reserves that underpin purposeful, health-sustaining behaviour [19]. Psychological resilience, conceptualised as a dynamic personal resource, reflects the ability to regulate emotions, adapt to adversity, and sustain goal-directed functioning under pressure [20]. When resilience is compromised by prolonged anxiety, individuals may struggle to initiate or maintain effortful behaviours, including PA.

Cognitive-behavioural and attentional control theories complement this resource-based account by specifying the mechanisms through which depletion occurs. Anxiety narrows attentional focus toward threat-related cues, impairs executive functioning, and undermines the self-regulatory capacity needed to initiate and maintain effortful behaviour [21]. Interpreted within the COR framework, these disruptions are not independent effects they are the visible manifestations of resource loss [22]. Together, they clarify how the loss of resilience as a psychological resource translates into the behavioural avoidance and motivational disengagement that reduces PA engagement.

This framework generates a theoretically coherent directional pathway: anxiety depletes psychological resources, resilience reflects the remaining adaptive capacity, and PA represents a goal-directed behaviour whose maintenance depends upon that capacity. Although the cross-sectional design of the present study precludes causal conclusions, this sequence is theoretically grounded, and the study examines whether the observed data are consistent with it. Longitudinal and experimental research will be needed to establish temporal precedence and confirm causal direction [23].

#### 1.1.1. Anxiety and Physical Activity

Anxiety is defined as an incessant worry, the increased sense of threat, and inability to control emotional reactions to stress [24]. Increased levels of anxiety among university students were also found to be negatively associated with decreased involvement in PA [25]. Behaviourally, anxiety promotes avoidance tendencies, reduces intrinsic motivation, and amplifies the subjective effort associated with physical exertion [26,27]. Students who have an increased level of anxiety can, thus, lose interest in PA as a method of distress reduction, although the positive impact of PA on mental health is well-established [28]. This relationship can be understood through several behavioural and cognitive pathways. Anxiety often promotes avoidance tendencies, diminishes intrinsic motivation, and amplifies the subjective effort required for physical exertion. Consequently, students experiencing heightened anxiety may withdraw from physical activity despite its well-documented mental and physical health benefits.

From an attentional control perspective, anxiety impairs the allocation of cognitive resources by prioritising threat-related processing over goal-directed action [21,29]. This paradox is particularly salient in academic environments, where chronic stress and limited recovery opportunities can reframe exercise as an additional demand rather than restorative practice. This cognitive interference makes it more difficult to plan, initiate, and sustain PA, especially when mental fatigue is already present. Together, these mechanisms help explain why anxiety frequently correlates with reduced participation in health-promoting behaviours.

#### 1.1.2. Role of Psychological Resilience

Psychological resilience is the ability to adjust to stress, overcome adversity, and maintain goal-directed behaviour in the face of stress [30,31]. It encompasses emotional regulation, cognitive flexibility, and behavioural persistence in the face of adversity [32,33]. Within the COR framework, it operates as a protective personal resource, one that enables individuals to absorb the impact of stressors without exhausting the reserves required for goal-directed behaviour [34]. However, when anxiety is chronic, it progressively erodes this capacity by heightening emotional reactivity, impairing attentional control, and depleting coping resources over time [19].

As resilience weakens, individuals become less equipped to sustain behaviours requiring consistent self-regulation, such as regular PA [2,35] Students with stronger resilience may remain physically active despite experiencing anxiety, drawing on emotional regulation and adaptive coping to manage distress without abandoning health-promoting behaviour [36,37]. Conversely, those whose resilience is depleted are more likely to disengage from goal-directed health behaviours when confronted with psychological strain [36,37,38].

This proposed pathway aligns with self-regulation theories, which emphasise that behavioural outcomes depend not only on external stressors but also on the internal psychological resources available to the individual [23]. The present study examines whether the observed data is consistent with this sequence, while acknowledging that cross-sectional evidence cannot establish causal direction. Longitudinal research will be necessary to confirm temporal precedence and test whether changes in resilience over time translate into changes in PA engagement [39].

## 2. Materials and Methods

### 2.1. Study Design

This cross-sectional study examined associations among anxiety, psychological resilience, and physical activity among university students in Punjab, Pakistan. All relationships are analysed as associations consistent with the proposed theoretical model, not as causal processes. The study a part of the Physical Activity and Wellbeing Study (PAWS), a broader research programme investigating psychological and behavioural correlates of PA in Pakistani populations. Data was collected in the period of May to December 2025 in the public and private universities, in six cities of the Punjab province Lahore, Faisalabad, Rawalpindi, Multan, Bahawalpur and Dera Ghazi Khan. These cities were selected to represent geographic and socioeconomic variation across north, central, and south Punjab, encompassing metropolitan, industrial, administrative, semi-urban, and less-developed regions.

### 2.2. Participants and Sampling

A multi-stage recruitment approach using non-probability sampling was adopted. In the first stage, 12 universities were purposively selected across six cities to ensure variation in geographic location, institutional type, and student enrolment size. Both public and private institutions were included to capture diversity in academic environments.

In the second stage, participants were recruited using convenience sampling within each institution. Students were approached in classrooms and common areas across different academic disciplines. Eligible participants were full-time undergraduate or master’s students aged 18 to 26 years who were enrolled at the time of data collection. Both male and female students were included.

### 2.3. Sample Size

Sample size was estimated using the standard single population proportion formula for cross sectional studies, n = (Z^2^ × p × (1 − p))/d^2^, as recommended by Lwanga and Lemeshow (1991) [40]. In the absence of reliable prevalence estimates for anxiety-related PA behaviour among Pakistani university students, the proportion (p) was conservatively set at 0.50 to maximise sample size [41,42]. A 95 percent confidence level (Z = 1.96) and a margin of error of 5 percent (d = 0.05) were applied.

Based on these assumptions, the minimum required sample size was 385 participants. To improve statistical power for mediation analysis and account for potential non-response and incomplete questionnaires, a target sample of approximately 1000 students was set. Of approximately 1000 students approached, 930 agreed to participate (response rate ≈ 93%). This larger sample also ensured adequate statistical power for mediation analysis [43] and sufficient class sizes for latent profile analysis [44].Following data screening, 160 questionnaires were excluded due to incomplete responses or evidence of careless responding (e.g., missing items exceeding 20% of scale items, or inconsistent response patterns), yielding a final analytical sample of 770 participants. Participants were recruited through in-person engagement across selected universities. All students who agreed to participate provided informed consent prior to completing the questionnaire. It should be noted that the use of convenience sampling, while appropriate for this exploratory study, limits generalizability to the broader Pakistani university population. The multi-city recruitment strategy was adopted specifically to partially mitigate this limitation by increasing geographic and institutional diversity.

### 2.4. Inclusion and Exclusion Criteria

The participants were selected based on the following criteria: between 18 and 26 years, full-time degree students, and capable of comprehending English language. Participants were excluded if they did not provide informed consent, submitted incomplete questionnaires, or demonstrated patterns indicative of careless or inattentive responding.

### 2.5. Ethics Approval

The research was carried out in compliance with ethical principles presented in the Declaration of Helsinki. The Bahauddin Zakariya University, Pakistan granted ethical approval (Approval Letter No: 112/UREC/2025). All participants were informed of the study and gave informed consent in written or verbal form before data collection.

### 2.6. Measures

**Figure 1:**
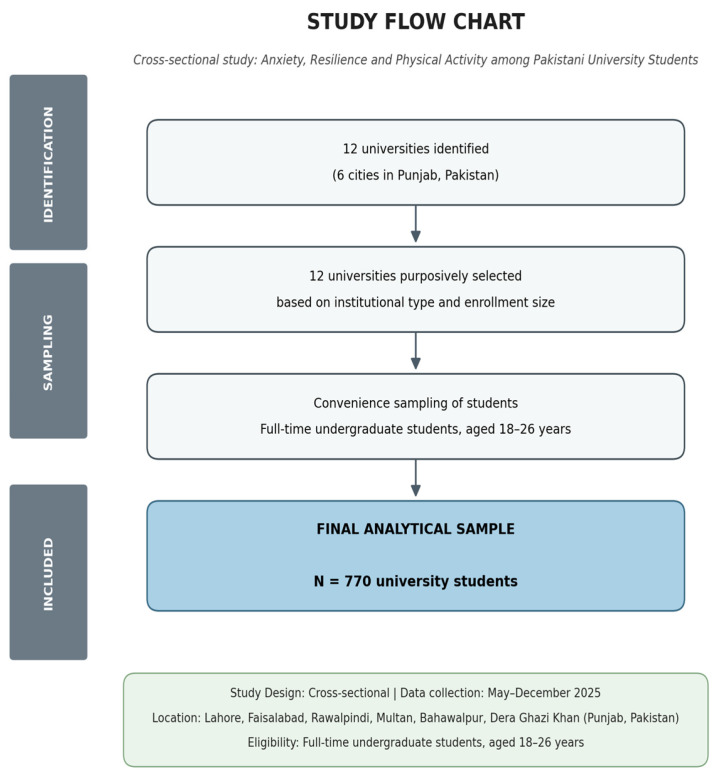
Flow chart of study procedure.

The survey included a demographic section and three validated scales. English versions of all instruments were used, as English is the primary medium of instruction and assessment in Pakistani universities. University students are therefore expected to possess adequate proficiency to comprehend and respond to questionnaire items reliably. Questionnaire administration was supervised by trained research assistants who were available to clarify any item-level confusion, reducing the risk of misinterpretation. Figure 1 shows study procedure. File S1 presents the questionnaire used for data collection in the current study.

#### 2.6.1. Demographic Characteristics

A structured demographic questionnaire assessed sociodemographic and academic variables. Participants self-reported age (continuous, in years), gender (male, female, or other/prefer not to say), university affiliation, academic major (categorized as Medical/Health Sciences, Engineering/Technical, Social Sciences/Humanities, Business/Commerce, Natural Sciences, or Other), and current semester of study (grouped as 1st–2nd Year, 3rd–4th Year, or 5th Year/Final). Marital status was recorded as single/never married, married, divorced/separated, or widowed.

Socioeconomic status was operationalized through monthly household income in Pakistani Rupees (PKR), categorized into five brackets based on World Bank lower-middle income economy thresholds for Pakistan: (0) no income; (1) PKR 6000–25,000 (approximately USD 38–151); (2) PKR 25,000–40,000 (USD 151–242); (3) PKR 40,000–80,000 (USD 242–484); and (4) PKR 80,000–170,000 (USD 484–1028) [13]. Residence type (urban vs. rural) was determined by self-reported city of residence, classified according to Pakistan Bureau of Statistics metropolitan classifications 2023. 

#### 2.6.2. Anxiety

Anxiety severity was assessed using the Generalized Anxiety Disorder 7-item scale (GAD-7), a validated self-report inventory based on DSM-IV diagnostic criteria [45]. The scale measures the frequency of anxiety symptoms in the last 2 weeks in seven domains: nervousness, worry, relaxation, difficulties, restlessness, irritability, and fear of awful events. Participants responded on a 4-point Likert scale (0 = Not at all to 3 = Nearly every day). Total scores range from 0 to 21, with higher scores indicating greater anxiety severity. Severity cutoffs were as follows: 0–4 (minimal), 5–9 (mild), 10–14 (moderate), and 15–21 (severe), with scores ≥10 indicating clinically significant anxiety.

The GAD-7 has demonstrated strong psychometric properties across diverse populations, with excellent internal consistency (Cronbach’s α = 0.92) and good overall reliability [45]. A cutoff score of 10 or higher has been shown to effectively identify generalized anxiety disorder, with high sensitivity (89%) and specificity (82%). In the current sample, the GAD-7 demonstrated excellent internal consistency (α = 0.90). The GAD-7 has also been translated into Urdu and validated in Pakistani populations, with evidence supporting its reliability and validity in both English and Urdu versions [46]. Although the Urdu version is available and culturally validated, the present study employed the English version of the scale, as English is the primary language of instruction and assessment in Pakistani universities.

#### 2.6.3. Psychological Resilience

Resilience was measured using the 10-item Connor-Davidson Resilience Scale (CD-RISC-10) [31,47]. The CD-RISC-10 assesses the ability to tolerate experiences such as change, personal problems, pressure, and failure across five core domains: adaptation, tenacity, recovery, emotional regulation, and self-efficacy. The scale consists of 10 items (e.g., “I am able to adapt when changes occur,” “I tend to bounce back after hardships,” “Under pressure, I stay focused”) rated on a 5-point Likert scale (0 = Not true at all to 4 = True nearly all the time), referencing the past month. Total scores range from 0 to 40, with higher scores indicating greater psychological resilience.

The CD-RISC-10 has shown robust psychometric properties across diverse populations, with internal consistency ranging from 0.85 to 0.92, including an initial validation coefficient of Cronbach’s α = 0.85 [47]. Test–retest reliability is also strong (r = 0.80) over four to six weeks (*p* < 0.001) [47]. Internal consistency in the current sample was excellent (α = 0.94). The scale is also translated into Urdu and proved to be used in Pakistani population to ensure semantic and conceptual equivalence [48]. Even though there is a validated Urdu version, the English version was applied in the current study due to its common use in the Pakistan higher education institutions.

#### 2.6.4. Physical Activity

Physical activity was measured by Cho, Physical Activity Scale [49], a culturally validated 5 item measure adapted to specific population groups, which includes Pakistan [13]. The Physical Activity Scale by Cho measures perceived habitual physical activity based on five items that measure: (1) frequency (How often do you engage in physical activity in your leisure time), (2) intensity, (3) duration, (4) type/nature of activity (sedentary to high-impact), (5) maintenance length (How long have you been maintaining this level). Each item is rated on a 5-point Likert scale (0 = Never/Lowest to 4 = Always/Highest), yielding a total score ranging from 0 to 20, where higher scores indicate higher self-reported PA engagement.

The scale has shown acceptable psychometric characteristics in Pakistani sample with test-retest reliability coefficients reported to be between 0.61 and 0.91 with some indication of convergent and discriminant validity evident [13]. The scale demonstrated excellent internal consistency in the present sample (α = 0.90). PA data were treated as a continuous score throughout all analyses. In the current research, English version was used, since it is widely used in the research and teaching at universities, and the presence of a validated Urdu version serves as an additional indication that the measure is culturally appropriate.

### 2.7. Data Collection Procedure

Data was collected using a mixed-mode approach. The main data collection strategy was the paper-based and in-person administration of questionnaires in the campuses of the university. This method was chosen in order to maximise the level of response since online-only surveys have been reported to attract a lower number of university students in Pakistan [13]. Questionnaires were administered through trained research assistants distributing them at scheduled class periods and in common student areas, by standardised administration procedures. To accommodate scheduling constraints and institutional access limitations, a secondary online survey option was used for a small proportion of participants. The online questionnaire replicated the paper-based version in content and structure. Participants accessing the online survey reviewed an information sheet and provided informed consent prior to participation. Completion time for both modes was approximately 10 min. All participants signed informed consent before data collection. Students were invited to take part and were notified that they could pull out at any time without consequence.

Data Management: Each completed questionnaire was screened for missing and inconsistent data. Responses with more than 20% missing items across any scale were excluded. For cases with missing values below this threshold, item mean substitution was applied within the affected scale, a standard procedure for Likert-type instruments with minimal missing data. All data was coded in Microsoft Excel and imported into SPSS 23 version for analysis. Total scores were computed by summing item responses according to each scale’s scoring guidelines. Data were checked for entry errors, out-of-range values, and distributional assumptions prior to analysis.

### 2.8. Statistical Analysis

Data were analyzed using SPSS version 23 and R Studio 4.3.0. Descriptive statistics summarised sociodemographic characteristics and scale scores as frequencies, percentages, and means with standard deviations. Independent-samples t-tests with Cohen’s d examined gender differences in anxiety, resilience, and PA; one-way ANOVAs with eta-squared (η^2^) examined differences across academic years. Pearson correlations with 95% confidence intervals (computed via Fisher z-transformation) examined bivariate associations among the three study variables. Hierarchical linear regression evaluated predictors of PA in three sequential steps: covariates only (gender and academic year; Model 1); addition of anxiety (Model 2); and addition of psychological resilience (Model 3). Unstandardised coefficients (B) with standard errors (SE), standardised coefficients (β), 95% confidence intervals, and incremental variance explained (ΔR^2^) are reported. Prior to regression analysis, distributional assumptions were verified: residuals were inspected for normality using Q-Q plots, homoscedasticity was assessed via residual plots, and multicollinearity was evaluated using variance inflation factors (VIF; acceptable threshold < 5). VIF values ranged from 1.001 to 1.292 across all predictors, indicating no multicollinearity concerns. Statistical significance was set at *p* < 0.05 throughout.

Mediation analysis examined whether psychological resilience mediated the association between anxiety and PA using the PROCESS macro (Model 4) in SPSS, with 5000 bootstrap resamples and bias-corrected confidence intervals. Path coefficients (a, b, c, c′) were estimated via multiple regression with gender and academic year as covariates. The indirect effect (a × b) was considered statistically significant when the 95% bootstrap confidence interval excluded zero. The proportion of total effect explained by the mediator was calculated as (indirect effect ÷ total effect) × 100.

Latent profile analysis (LPA) was conducted to identify distinct subgroups of students based on their co-occurring patterns of anxiety, resilience, and PA. LPA is a person-centred technique that classifies individuals into mutually exclusive latent classes based on continuous indicator profiles and was selected because it directly captures the joint patterning of the three constructs within individuals, aligning with the study’s secondary objective. Models specifying one to six latent classes were estimated and compared using five complementary fit criteria: (1) the Akaike information criterion (AIC) and (2) Bayesian information criterion (BIC), where lower values indicate better fit; (3) the sample-size-adjusted BIC (saBIC); (4) relative entropy (range 0–1; values ≥ 0.70 indicate adequate classification accuracy) [50]; and (5) the Lo–Mendell–Rubin adjusted likelihood ratio test (LMR-LRT) [51], which tests whether a K-class solution fits significantly better than a K−1 solution.

The optimal class solution was selected based on convergence of four pre-specified criteria: (1) lowest or near-lowest BIC and saBIC with meaningful improvement over the preceding solution (ΔBIC ≥ 10) [52]; (2) entropy ≥ 0.70 confirmed by mean posterior classification probabilities ≥ 0.80 per class; (3) class-size adequacy, with each class comprising at least 5% of the total sample [44]; and (4) theoretical interpretability, each profile being conceptually distinct and substantively meaningful within the study’s framework. Once profiles were identified, one-way ANOVAs with Bonferroni-corrected post-hoc comparisons (adjusted α = 0.017) and eta-squared effect sizes examined differences in anxiety, resilience, and PA across classes.

## 3. Results

Table 1 presents the demographic and socioeconomic characteristics of the study participants (N = 770). The study included 770 university students aged 18–26 years (mean age = 21.48 ± 2.10 years). The sample was gender balanced, comprising 51.4% males and 48.6% females. Participants were evenly distributed across academic years, with representation from Year 1 (27.8%) through Year 4 (20.5%). Most students were enrolled in public sector universities (59.9%). Participants were recruited from multiple cities across Punjab, with the largest proportions from Lahore (24.5%), Faisalabad (20.3%), and Multan (19.2%). The majority were single (83.2%). More than half of the sample reported no personal monthly income (58.8%), while the remainder reported predominantly low to moderate income levels.

Table 2 summarises descriptive statistics and group comparisons for the three study scales. Overall, mean anxiety was 6.65 (SD = 5.88), mean resilience was 17.92 (SD = 10.63), and mean PA was 9.19 (SD = 5.96). No statistically significant differences were observed by gender for anxiety (t = 0.672, *p* = 0.502, d = 0.05), resilience (t = −0.570, *p* = 0.569, d = −0.04), or PA (t = −0.031, *p* = 0.975, d = −0.00). Similarly, one-way ANOVAs revealed no significant differences across academic years for anxiety (F(3, 766) = 0.816, *p* = 0.485, η^2^ = 0.003), resilience (F(3, 766) = 0.526, *p* = 0.665, η^2^ = 0.002), or PA (F(3, 766) = 0.351, *p* = 0.789, η^2^ = 0.001). All effect sizes were negligible across gender and year groups, confirming that these variables are appropriate statistical controls rather than substantive predictors.

**Figure 2:**
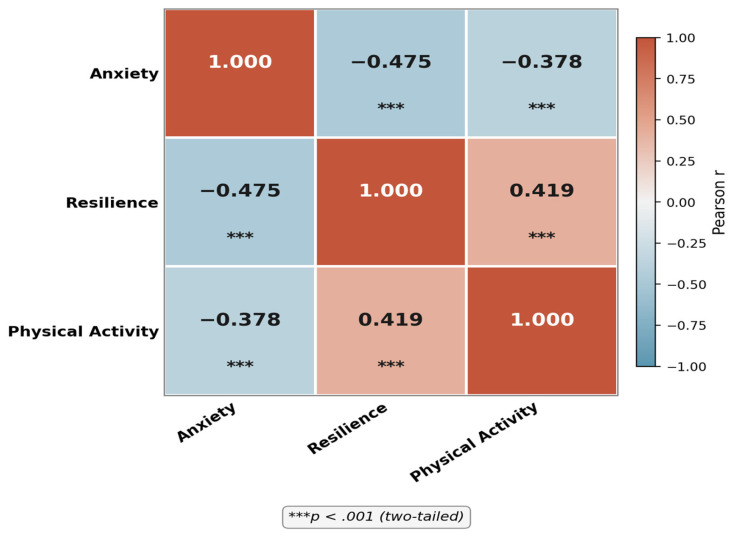
Pearson correlation matrix of study variables (anxiety, psychological resilience, and PA).

Figure 2 illustrates the Pearson correlation matrix among the three study variables. Anxiety was significantly and negatively correlated with psychological resilience (r = −0.475, *p* < 0.001, 95% CI [−0.528, −0.418]) and with PA (r = −0.378, *p* < 0.001, 95% CI [−0.437, −0.315]), indicating that higher anxiety was associated with both lower resilience and reduced PA engagement. Psychological resilience showed a significant positive association with PA (r = 0.419, *p* < 0.001, 95% CI [0.359, 0.476]), such that students with greater resilience tended to report higher PA levels. All three correlations were statistically significant and in the expected directions, providing preliminary support for the hypothesised mediation pathway.

Table 3 presents the hierarchical regression analysis predicting PA. Gender and academic year (Model 1) explained a negligible and non-significant proportion of variance (R^2^ = 0.001), confirming their suitability as covariates. The addition of anxiety in Model 2 produced a substantial improvement in fit (ΔR^2^ = 0.142, *p* < 0.001), with anxiety emerging as a significant negative predictor (β = −0.377, *p* < 0.001). Psychological resilience, added in Model 3, contributed a further significant increment (ΔR^2^ = 0.074, *p* < 0.001) and was a significant positive predictor (β = +0.310, *p* < 0.001). Anxiety remained significant but attenuated (β = −0.230, *p* < 0.001), consistent with partial mediation. The final model explained 21.7% of variance in PA (adjusted R^2^ = 0.213). Full coefficient details are presented in Table 3.

Table 4 presents the mediation analysis. Anxiety was significantly and negatively associated with psychological resilience (path a: B = −0.858, SE = 0.058, *p* < 0.001). Resilience was, in turn, positively associated with PA after controlling for anxiety (path b: B = 0.174, SE = 0.020, *p* < 0.001). The total effect of anxiety on PA was significant (path c: B = −0.383, SE = 0.034, *p* < 0.001). When resilience was included as a mediator, the direct effect of anxiety on PA remained significant but was reduced in magnitude (path c′: B = −0.234, SE = 0.037, *p* < 0.001), consistent with partial mediation. The indirect effect was significant (a × b = −0.149, SE = 0.021, 95% bootstrap CI [−0.189, −0.109]), as the confidence interval excluded zero. Psychological resilience accounted for approximately 39.0% of the total effect of anxiety on PA, indicating that resilience functions as a meaningful but partial mechanism through which anxiety inhibits physical activity engagement.

**Figure 3:**
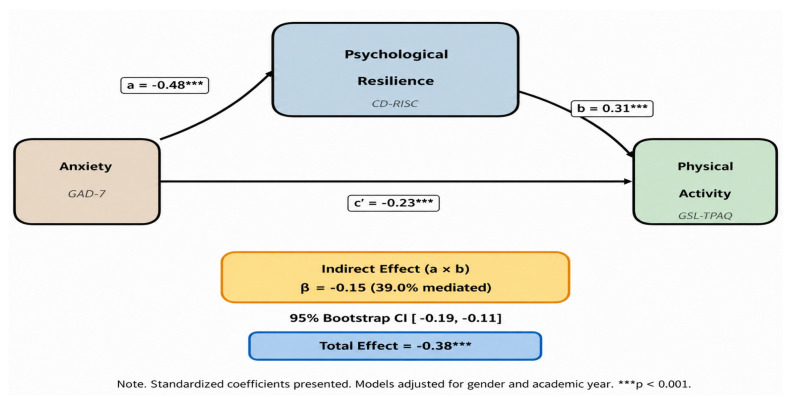
Mediation model illustrates the role of psychological resilience in the association between anxiety and physical activity. Standardized coefficients (β) are presented. The indirect effect was significant based on bootstrap resampling (5000 samples), with the 95% confidence interval not including zero.

Figure 3 illustrates the mediation pathway linking anxiety, psychological resilience, and physical activity. In the standardized model, anxiety showed a moderate negative association with psychological resilience (β = −0.48, *p* < 0.001), while psychological resilience was positively associated with physical activity (β = 0.31, *p* < 0.001). Anxiety also retained a direct negative association with physical activity (β = −0.23, *p* < 0.001), although this effect was weaker than the total effect (β = −0.38, *p* < 0.001). The indirect effect (β = −0.15, 95% CI [−0.19, −0.11]) accounted for approximately 39.0% of the total effect. Together, these results indicate that higher anxiety is linked to lower resilience, which in turn relates to lower physical activity, visually confirming the partial mediating role of psychological resilience. 

LPA models specifying one to six latent classes were estimated and compared using multiple fit indices (Table 5). Information criteria (AIC, BIC, and saBIC) decreased substantially from K = 1 to K = 3 (ΔBIC1→2 = −187.68; ΔBIC2→3 = −62.60), indicating meaningful improvement with each additional class. Beyond K = 3, improvements fell below the recommended ΔBIC ≥ 10 threshold, and the six-class solution produced a BIC reversal (ΔBIC = +11.97), indicating model deterioration. The LMR-LRT was statistically significant across all transitions (all p < 0.001). The three-class solution yielded adequate classification accuracy (entropy = 0.729), with mean posterior classification probabilities of 0.904, 0.867, and 0.844 for Classes 1, 2, and 3 respectively, all exceeding the recommended 0.80 threshold. The three-class solution was therefore retained as the most statistically adequate, parsimonious, and theoretically interpretable solution.

Table 6 presents the descriptive statistics for the three latent profiles. The Anxious–Inactive profile (Class 1; n = 209, 27.1%) was characterised by clinically elevated anxiety (M = 14.48, SD = 3.49; moderate–severe range on the GAD-7), markedly low resilience (M = 8.51, SD = 6.99), and the lowest PA engagement in the sample (M = 6.18, SD = 5.36). The Moderately Distressed–Moderately Active profile (Class 2; n = 362, 47.0%)—the largest class, representing nearly half the sample, displayed mild-to-moderate anxiety (M = 5.49, SD = 2.97), intermediate resilience (M = 18.82, SD = 9.22), and moderate PA (M = 8.86, SD = 5.62). The Resilient–Active profile (Class 3; n = 199, 25.8%) was distinguished by minimal anxiety (M = 0.54, SD = 0.66), the highest resilience (M = 26.17, SD = 8.33), and the highest PA engagement (M = 12.95, SD = 5.12). A coherent gradient was evident across all three constructs: as class-level PA increased from Class 1 to Class 3, anxiety decreased and resilience increased progressively, providing person-centred corroboration of the variable-centred mediation findings.

Table 7 presents the one-way ANOVA results and Bonferroni-corrected post-hoc comparisons across the three latent profiles. All three omnibus effects were statistically significant and large in magnitude. For anxiety, F(2, 767) = 1367.84, *p* < 0.001, η^2^ = 0.781, indicating that class membership accounted for 78.1% of the variance in anxiety scores—a very large effect. Bonferroni-corrected comparisons confirmed that all three classes differed significantly from one another (all p < 0.001). For psychological resilience, F(2, 767) = 227.14, *p* < 0.001, η^2^ = 0.372, with all pairwise comparisons significant (all p < 0.001), confirming the progressive depletion of resilience resources from the Resilient–Active to the Anxious–Inactive profile. For physical activity, F(2, 767) = 80.83, *p* < 0.001, η^2^ = 0.174, with all three classes differing significantly from one another (all p < 0.001). Together, these results confirm that the three latent profiles are statistically distinct and exhibit a coherent, monotonic gradient in which increasing anxiety is systematically associated with diminishing resilience and lower PA engagement.

**Figure 4:**
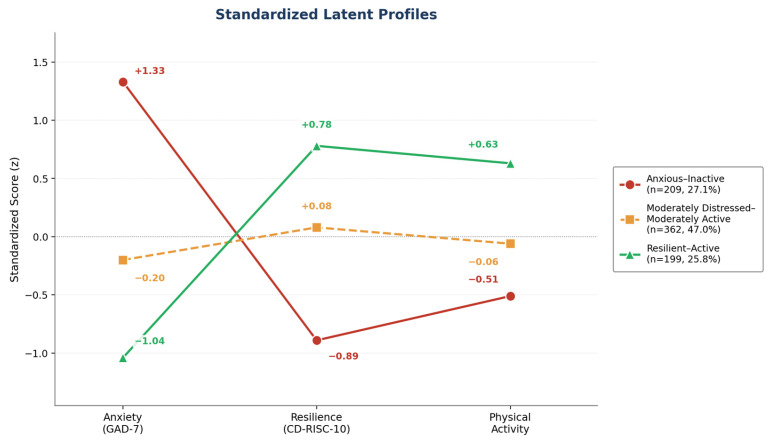
Standardised latent profiles of anxiety, psychological resilience, and physical activity across the three-class solution. Class 1 = Anxious–Inactive (n = 209, 27.1%); Class 2 = Moderately Distressed–Moderately Active (n = 362, 47.0%); Class 3 = Resilient–Active (n = 199, 25.8%). A clear dose response gradient is evident, with anxiety decreasing and resilience increasing progressively from Class 1 to Class 3.

Figure 4 illustrates the standardized profiles of anxiety, psychological resilience, and physical activity across the three PA groups. The figure highlights a clear dose–response pattern, with anxiety decreasing and resilience increasing progressively from the Low PA to the High PA profile. Together, the table and figure indicate that higher engagement in physical activity is associated with lower anxiety and greater psychological resilience, providing person-centred support for the variable-centred mediation findings.

## 4. Discussion

This study examined the associations among anxiety, psychological resilience and PA in a sample of Pakistani university students. Consistent with the hypothesised pattern, anxiety was negatively associated with both resilience and PA, while resilience showed a positive association with PA. Together, anxiety and resilience explained 21.7% of the variance in PA. Furthermore, mediation analysis revealed that psychological resilience as a partial mediator of the anxiety–PA association, with the indirect pathway through resilience accounting for approximately 39% of the total statistical effect. Latent profile analysis further identified three distinct subgroups, ranging from a profile characterized by high anxiety and low resilience alongside minimal PA, to one marked by low anxiety, high resilience, and greater PA engagement.

### 4.1. Role of Anxiety and Physical Activity

The negative association between anxiety and PA observed in this study is consistent with a well-established pattern in literature. Prior studies across Europe, East Asia, and North America have similarly found anxiety to be linked with lower activity frequency, reduced exercise adherence, and increased behavioural avoidance [53,54]. Studies using the GAD-7 specifically have also reported elevated anxiety symptomatology among physically inactive university cohorts [55]. The present findings extend this evidence to a Pakistani university population, where structural and cultural constraints further limit opportunities for PA. Students with higher anxiety levels showed lower PA participation, a pattern consistent with avoidance-based behavioural responses and impaired self-regulation [56].

From a theoretical standpoint, this association is interpretable through attentional control theory, which suggests that anxiety narrows the cognitive resources available for initiating and sustaining goal-directed behaviour [57,58]. In a setting where mean GAD-7 scores approached the mild-to-moderate threshold (M = 6.65), it is plausible that students perceived physical exertion as an additional deman rather than a restorative outlet, a pattern noted in similar cross-sectional research [59]. Anxiety alone was associated with 14.3% of the variance in PA, indicating that it represents a meaningful but partial barrier operating alongside other psychosocial factors, most notably resilience. This is imprtant in the Pakistani context, where high academic demands intersect with limited recreational infrastructure and restricted access to organized PA opportunities [60,61,62], conditions that may make it especially difficult for anxious students to remain physically active precisely when doing so would be most beneficial.

### 4.2. The Mediating Role of Psychological Resilience

The data were consistent with psychological resilience as a partial mediator of the anxiety–PA association. Higher anxiety was associated with lower resilience, which in turn was associated with reduced PA engagement, a pattern aligned with cognitive-behavioural and self-regulation models suggesting that anxiety may weaken behavioural persistence by impairing emotional regulation, self-efficacy, and adaptive coping [30,34].

Resilience was statistically associated with approximately 39% of the total anxiety–PA relationship. This proportion should be interpreted cautiously: it reflects the extent to which the anxiety–PA association is accounted for by resilience within this cross-sectional design, rather than a causal quantity. This estimate is comparable to, and in some cases exceeds, those reported in recent studies among university students in other regions [63], suggesting that psychological resources play a central role in sustaining health behaviours under stress across diverse contexts.

In university populations, higher resilience has been associated with greater behavioural flexibility and sustained engagement in PA despite academic and social stressors [64]. The present findings extend this evidence to a South Asian population, highlighting the particular importance of resilience in low- and middle-income settings where access to formal mental health services is limited and students rely more heavily on personal coping resources [65,66]. This is further consistent with perspectives framing resilience as a dynamic and developable capacity rather than a fixed trait, one that may buffer the association between emotional distress and behavioural disengagement [28].

In Pakistan university students specifically, anxiety may impair attentional control, executive functioning, and sleep quality, thereby eroding the psychological resources necessary for maintaining regular PA [13]. The observed indirect pathway suggests that resilience may represent a meaningful point of focus in intervention design within resource-constrained settings. Rather than concentrating exclusively on anxiety reduction, which typically requires specialised clinical services that remain scarce in Pakistan, approaches that also target resilience development may be worth exploring alongside conventional support. Group-based skills programmes, peer support networks, and PA initiatives incorporating mindfulness components represent possible directions, though their effectiveness in this specific context would need to be evaluated through experimental research.

### 4.3. Person-Centred Patterns of Physical Activity and Psychological Functioning

Beyond variable-centred associations, the latent profile analysis identified three distinct subgroups that differed meaningfully in their co-occurring patterns of anxiety, resilience, and PA. The value of this person-centred approach lies not in replicating the correlation findings, but in demonstrating that these constructs cluster together within identifiable student subgroups, something average associations alone cannot reveal. Consistent with prior person-centred research in student populations [67,68], low-activity students were not simply less active; they were simultaneously more anxious and less resilient, suggesting a coherent psychological profile rather than isolated trait differences [69].

The graded gradient across the three classes, with anxiety decreasing and resilience increasing progressively from Class 1 to Class 3 is theoretically interpretable within the COR framework. Students in the Anxious–Inactive profile may disengage from PA partly as a short-term strategy to conserve depleted psychological resources, even at the cost of longer-term health [18]. The Moderately Distressed–Moderately Active group, representing nearly half the sample, is of particular interest: these students are neither acutely distressed nor optimally functioning, and may represent a prevention opportunity, a group whose trajectories could shift in either direction depending on the demands they face and the resources available to them. The Resilient–Active profile, by contrast, illustrates that sustained PA engagement is possible even within a high-pressure university environment, when adequate psychological resources are present.

### 4.4. Implications

These findings have potential implications for university-based health promotion in resource-constrained settings, though the cross-sectional design means these should be understood as possible directions rather than evidence-based prescriptions.

Given that resilience was statistically associated with approximately 39% of the anxiety–PA relationship, approaches that combine anxiety support with resilience-building may be worth exploring alongside exclusive clinical intervention. In Pakistani universities, where counselling services are limited and mental health stigma remains prevalent [13], strength-based resilience programming may be more accessible and acceptable than pathology-focused approaches. Brief skills-based interventions targeting coping, emotional regulation, and self-efficacy have shown promise in related contexts [70], though their effectiveness in this specific setting would need to be evaluated.

The Moderately Distressed–Moderately Active group (47.0% of the sample) may represent a useful focus for upstream, preventive approaches. These students are not acutely distressed but could be vulnerable to deterioration under sustained academic pressure [71,72]. Low-cost campus initiatives such as peer-led activity groups or curriculum-integrated wellbeing components might potentially support resilience and PA engagement before clinical thresholds are reached, though this remains to be tested. For students in the Anxious–Inactive profile (27.1%), where high anxiety and low resilience co-occur, more intensive and targeted approaches addressing both psychological and behavioural dimensions simultaneously may be worth considering. It is important that any such profile-informed thinking guides supportive and inclusive programming rather than serving to categorise or stigmatise individuals.

The absence of gender and academic year differences in anxiety, resilience, and PA is worth noting. Rather than treating these as surprising findings, they may reflect the specific structure of this sample or broader contextual features of Pakistani higher education, where anxiety pressures may be distributed relatively evenly across gender and year groups, possibly beginning early and persisting throughout the degree [73]. The high proportion of students reporting no personal income (58.8%) further highlights economic vulnerability as a contextual factor that may compound psychological distress and limit access to PA opportunities independently of the variables examined here.

### 4.5. Strengths, Limitations and Future Direction

This study has several strengths worth acknowledging. The sample size was adequate for both mediation analysis and latent profile analysis, and recruitment across six cities in Punjab improved geographic representativeness relative to single-institution designs. The use of validated, culturally appropriate instruments and the combination of variable-centred and person-centred analytic methods represent methodological contributions that go beyond what either approach alone could offer in this context.

Several limitations should be considered when interpreting these findings. Most importantly, the cross-sectional design means that temporal precedence cannot be established. The proposed pathway anxiety associated with lower resilience, which in turn is associated with reduced PA is theoretically grounded, but reverse and bidirectional associations are equally plausible. It is just as conceivable that physical inactivity contributes to anxiety, or that resilience influences how anxiety develops over time. Longitudinal research is needed to examine the directionality of these associations. PA was measured exclusively through self-report, which is susceptible to recall bias and social desirability, and may overestimate actual activity levels. Objective measurement using accelerometry would strengthen future work. The use of English-language instruments, while appropriate given the university context, may have introduced subtle comprehension variation among participants with lower English proficiency. Convenience sampling, despite the multi-city approach, limits generalisability to the broader Pakistani university population. Finally, other potentially relevant variables including sleep quality, social support, and academic workload were not measured and may account for additional variance in the observed associations.

Future research should prioritise longitudinal designs to establish whether changes in resilience over time are associated with changes in PA among anxious students, particularly during high-stress transition periods such as first-year enrolment. Experimental studies testing resilience-based interventions alongside PA promotion would provide more direct evidence for the practical implications suggested by these findings.

## 5. Conclusions

This study examined associations among anxiety, psychological resilience, and physical activity in Pakistani university students. The findings were consistent with a model in which resilience partially accounts for the anxiety–PA association, with the indirect pathway through resilience representing approximately 39% of the total statistical association. Latent profile analysis identified three distinct subgroups: an Anxious–Inactive profile (27.1%), a Moderately Distressed–Moderately Active profile (47.0%), and a Resilient–Active profile (25.8%), differing meaningfully in their co-occurring patterns of anxiety, resilience, and PA.

These findings suggest that resilience may be a meaningful correlate of PA engagement where anxiety is elevated and formal support is limited. For students in the Anxious–Inactive subgroup, integrated approaches combining cognitive-behavioural support with structured PA may be worth exploring. For the moderate group, lighter preventive strategies such as peer-led activity programmes or brief resilience workshops represent possible directions. These remain hypotheses to be tested in future longitudinal and experimental research rather than established recommendations and should be interpreted within the constraints of a cross-sectional, self-report design conducted in a specific South Asian university context.

## Figures and Tables

**Table 1:** Characteristics of the study participants sample (N = 770).

Characteristic	n (%) or M ± SD
Age (18–26 years)	21.48 ± 2.10
Gender	Male 396 (51.4%); Female 374 (48.6%)
Year of study (Y1–Y4)	Y1: 214 (27.8%); Y2: 210 (27.3%); Y3: 188 (24.4%); Y4: 158 (20.5%)
University sector	Public 461 (59.9%); Private 309 (40.1%)
City distribution	Lahore: 189 (24.5%); Faisalabad: 156 (20.3%); Multan: 148 (19.2%); Bahawalpur: 118 (15.3%); Rawalpindi: 111 (14.4%); Dera Ghazi Khan: 48 (6.2%)
Marital status	Single/Never Married 641 (83.2%); Married 123 (16.0%); Divorced/Separated 6 (0.8%)
Monthly income (PKR)	No income: 453 (58.8%); 6–25 k: 205 (26.6%); 25–40 k: 72 (9.4%); 40–80 k: 29 (3.8%); 80–170 k: 11 (1.4%)

**Note:** Values are presented as n (%) unless stated otherwise. Age is presented as mean ± SD and range. N reflects complete valid cases included in the analysis. PKR = Pakistani Rupees; USD conversions based on exchange rates at time of data collection (1 USD ≈ 158 PKR).

**Table 2:** Descriptive Statistics for Anxiety, Psychological Resilience, and Physical Activity (N = 770).

Variable/Group	n (%)	GAD-7 M ± SD	CDR-10 M ± SD	PA M ± SD
Overall	770 (100%)	6.65 ± 5.88	17.92 ± 10.63	9.19 ± 5.96
Gender				
Male	396 (51.4%)	6.79 ± 5.74	17.71 ± 10.24	9.18 ± 5.95
Female	374 (48.6%)	6.51 ± 6.03	18.14 ± 11.04	9.20 ± 5.98
*t*-test		0.672	−0.570	−0.031
*p*-value		0.502	0.569	0.975
Cohen’s d		0.05	−0.04	−0.00
Academic Year				
Freshmen (Y1)	214 (27.8%)	6.61 ± 5.79	18.25 ± 10.70	9.29 ± 5.99
Sophomore (Y2)	210 (27.3%)	6.19 ± 5.66	18.07 ± 10.17	9.44 ± 5.96
Junior (Y3)	188 (24.4%)	6.84 ± 5.84	18.16 ± 10.50	9.11 ± 5.77
Senior (Y4)	158 (20.5%)	7.10 ± 6.24	16.98 ± 11.20	8.82 ± 6.10
ANOVA F(3766)		0.816	0.526	0.351
*p*-value		0.485	0.665	0.789
η^2^		0.003	0.002	0.001

**Note:** Values are presented as M ± SD. GAD-7 = Generalized Anxiety Disorder 7-item Scale (total score 0–21); CD-RISC-10 = Connor-Davidson Resilience Scale 10-item (total score 0–40); PA = Physical Activity, Cho Scale (total score 0–20). All effect sizes were negligible across gender (Cohen’s d ≤ 0.05) and academic year (η^2^ ≤ 0.003).

**Table 3:** Hierarchical Regression Analysis Examining Associations of Anxiety and Psychological Resilience with PA.

Predictor	Model 1			Model 2		Model 3		
	B (SE)	β	95% CI	B (SE)	β	B (SE)	β	95% CI
Gender	0.019 (0.430)	0.002	[−0.83, 0.86]	−0.093 (0.399)	−0.008	−0.127 (0.382)	−0.011	[−0.88, 0.62]
Academic Year	−0.164 (0.196)	−0.030	[−0.55, 0.22]	−0.086 (0.182)	−0.016	−0.056 (0.174)	−0.010	[−0.40, 0.29]
Anxiety (GAD-7)	—	—	—	−0.383 (0.034) ***	−0.377 ***	−0.234 (0.037) ***	−0.230 ***	[−0.31, −0.16]
Resilience (CDR-10)	—	—	—	—	—	0.174 (0.020) ***	0.310 ***	[0.13, 0.21]
R^2^	0.001			0.143		0.217		
Adjusted R^2^	−0.002			0.140		0.213		
ΔR^2^	—			0.142 ***		0.074 ***		
F (model)	0.35			42.56 ***		53.08 ***		

**Note:** N = 770. Dependent variable: Physical Activity total score (Cho Scale, 0–20). B = unstandardised coefficient; SE = standard error; β = standardised coefficient; 95% CI = bias-corrected confidence interval for B. Gender coded 0 = Male, 1 = Female. Academic Year coded 1 = Freshmen to 4 = Senior. Model 1: F(2, 767); Model 2: F(3, 766); Model 3: F(4, 765). VIF range 1.001–1.292 (no multicollinearity). Durbin-Watson = 2.052. Zero standardised residuals exceeded 3.0. Highlighted green column = final model. *** *p* < 0.001.

**Table 4:** Mediation analysis examining psychological resilience as a mediator of the association between anxiety and PA.

Path	Description	B	SE	95% CI	*p*
a	Anxiety → Resilience	−0.86	0.06	[−0.97, −0.75]	<0.001
b	Resilience → Physical Activity (controlling Anxiety)	0.17	0.02	[0.13, 0.21]	<0.001
c	Total effect: Anxiety → Physical Activity	−0.38	0.03	[−0.45, −0.32]	<0.001
c′	Direct effect: Anxiety → Physical Activity (controlling Resilience)	−0.23	0.04	[−0.31, −0.16]	<0.001
a × b (indirect effect)	Anxiety → Resilience → Physical Activity	−0.15	0.02	[−0.19, −0.11]	<0.001

**Note:** N = 770. B = unstandardized regression coefficient. SE = standard error from bootstrap distribution (5000 resamples). 95% CI = bias-corrected bootstrap confidence interval. The indirect effect is significant because the CI does not include zero. c′ remains significant (*p* < 0.001), confirming partial (not full) mediation. All models adjusted for gender and academic year.

**Table 5:** Latent Profile Analysis Model Fit Indices for K = 1–6 Classes (N = 770).

K	Log-Likelihood	AIC	BIC	saBIC	Entropy	ΔBIC	LMR *p*
1	−3085.12	6188.24	6230.05	6201.47	—	—	—
2	−2958.05	5954.09	6042.37	5982.04	0.666	−187.68	<0.001
**3 ◄**	**−2893.52**	**5845.03**	**5979.78**	**5887.69**	**0.729**	**−62.60**	**<0.001**
4	−2833.37	5744.74	5925.95	5802.10	0.779	−53.83	<0.001
5	−2781.95	5661.90	5889.57	5733.98	0.734	−36.37	<0.001
6	−2754.70	5627.41	5901.54	5714.19	0.752	**+11.97**	<0.001

**Note:** ◄ = selected 3-class solution. AIC = Akaike Information Criterion; BIC = Bayesian Information Criterion; saBIC = sample-size-adjusted BIC; Entropy ≥ 0.70 indicates adequate classification accuracy. ΔBIC = change in BIC from prior solution; ΔBIC ≥ 10 indicates meaningful improvement. K = 6 shows BIC reversal (+11.97), indicating model deterioration. The K = 4–5 solutions showed indistinct profiles and were excluded on interpretability grounds.

**Table 6:** Three-Class Latent Profile Solution.

Profile	n (%)	Anxiety Mean ± SD	Resilience Mean ± SD	PA Mean ± SD	Post. Prob.
Class 1: Anxious–Inactive	209 (27.1%)	14.48 (3.49)	8.51 (6.99)	6.18 (5.36)	0.904
Class 2: Moderately Distressed–Moderately Active	362 (47.0%)	5.49 (2.97)	18.82 (9.22)	8.86 (5.62)	0.867
Class 3: Resilient–Active	199 (25.8%)	0.54 (0.66)	26.17 (8.33)	12.95 (5.12)	0.844

**Note:** Overall entropy = 0.729, indicating adequate classification accuracy. Post. Prob. = mean posterior classification probability per class (all ≥ 0.84). Colour coding: Red = highest anxiety/lowest resilience; Amber = intermediate; Green = lowest anxiety/highest resilience. A clear gradient is evident: as class-level PA increases, anxiety decreases and resilience increases progressively.

**Table 7:** ANOVA and Post-Hoc Comparisons Across LPA Classes.

Variable	F (df1, df2)	*p*	η^2^	Bonferroni Post-Hoc Comparisons
Anxiety (GAD-7)	1367.84	<0.001	0.781	C1 > C2: t = 32.61, *p* < 0.001 *** C1 > C3: t = 55.37, *p* < 0.001 *** C2 > C3: t = 23.19, *p* < 0.001 ***
Resilience (CDR-10)	227.14	<0.001	0.372	C1 < C2: t = −14.01, *p* < 0.001 *** C1 < C3: t = −23.24, *p* < 0.001 *** C2 < C3: t = −9.33, *p* < 0.001 ***
Physical Activity	80.83	<0.001	0.174	C1 < C2: t = −5.58, *p* < 0.001 *** C1 < C3: t = −13.04, *p* < 0.001 *** C2 < C3: t = −8.52, *p* < 0.001 ***

**Note:** F(2, 767) for all variables. η^2^ = eta-squared effect size (small ≥ 0.01; medium ≥ 0.06; large ≥ 0.14). All three effects are large. Bonferroni correction applied (α = 0.05/3 = 0.017). C1 = Anxious–Inactive (n = 209); C2 = Moderately Distressed–Moderately Active (n = 362); C3 = Resilient–Active (n = 199). All pairwise comparisons significant at *p* < 0.001. *** *p* < 0.001.

## Data Availability

Data can be requested by contacting the corresponding author on a reasonable request.

## Supplementary Materials

The following supporting information can be downloaded at: https://www.mdpi.com/article/10.3390/healthcare14091246/s1, File S1: Health and Well-being Survey.

## References

[1] McCloud T., Kamenov S., Callender C., Lewis G., Lewis G. (2023). The association between higher education attendance and common mental health problems among young people in England: Evidence from two population-based cohorts. Lancet Public Health.

[2] Campbell F., Blank L., Cantrell A., Baxter S., Blackmore C., Dixon J., Goyder E. (2022). Factors that influence mental health of university and college students in the UK: A systematic review. BMC Public Health.

[3] Sivertsen B., Hysing M., Knapstad M., Harvey A.G., Reneflot A., Lønning K.J., O’Connor R.C. (2019). Suicide attempts and non-suicidal self-harm among university students: Prevalence study. BJPsych Open.

[4] Wijs L.A., Doherty D.A., Keelan J.A., Burton P., Yovich J.L., Robinson M., Hart R.J. (2022). Mental health and behavioural problems in adolescents conceived after ART. Hum. Reprod..

[5] DiFonte M.C., Schick M.R., Spillane N.S. (2024). Perceived stress and resilience among college students: The roles of self-compassion and anxiety symptomatology. J. Am. Coll. Health.

[6] Fagaras S.-P., Radu L.-E., Vanvu G. (2015). The Level of Physical Activity of University Students. Procedia-Soc. Behav. Sci..

[7] Strain T., Flaxman S., Guthold R., Semenova E., Cowan M., Riley L.M., Bull F.C., Stevens G.A. (2024). National, regional, and global trends in insufficient physical activity among adults from 2000 to 2022: A pooled analysis of 507 population-based surveys with 5·7 million participants. Lancet Glob. Health.

[8] Brinkhof L.P., Ridderinkhof K.R., Murre J.M., Krugers H.J., de Wit S. (2023). Improving goal striving and resilience in older adults through a personalized metacognitive self-help intervention: A protocol paper. BMC Psychol..

[9] Southwick S.M., Bonanno G.A., Masten A.S., Panter-Brick C., Yehuda R. (2014). Resilience definitions, theory, and challenges: Interdisciplinary perspectives. Eur. J. Psychotr..

[10] Yang F., Gao Y., Liu F. (2025). Physical exercise and depression in university students with psychological resilience as mediator and family support as moderator. Sci. Rep..

[11] Lei G., Li L., Zhang L. (2025). Mental health literacy and anxiety in college students mediating role of psychological resilience and moderating role of physical exercise. Sci. Rep..

[12] Pirwani N., Szabo A. (2024). Could physical activity alleviate smartphone addiction in university students? A systematic literature review. Prev. Med. Rep..

[13] Kayani S., Wang J., Biasutti M., Zagalaz Sánchez M.L., Kiyani T., Kayani S. (2020). Mechanism between physical activity and academic anxiety: Evidence from Pakistan. Sustainability.

[14] Hadier S.G., Yinghai L., Long L., Hamdani S.D., Hamdani S.M.Z.H. (2025). Assessing physical literacy and establishing normative reference curves for 8–12-year-old children from South Punjab, Pakistan: The PAK-IPPL cross-sectional study. PLoS ONE.

[15] Rehman R., Fatima K., Hussain M., Sarim M., Gazzaz Z.J., Baig M. (2021). Association between depression and health risk behaviors among university students, Karachi, Pakistan. Cogent Psychol..

[16] Azim S.R., Adnan N., Azim S.N., Nisar M., Shamim M.S. (2022). Frequency of mental distress among medical students from selected medical colleges of Pakistan: A systematic review. J. Pak. Med. Assoc..

[17] Thomas C.L., Nair M., Ganske J.K. (2024). Coping Profiles and Mental Health Outcomes in American University Students: A Latent Profile Analysis. Psychol. Rep..

[18] Hobfoll S.E. (1989). Conservation of resources: A new attempt at conceptualizing stress. Am. Psychol..

[19] Hobfoll S.E. (2001). The Influence of Culture, Community, and the Nested-Self in the Stress Process: Advancing Conservation of Resources Theory. Appl. Psychol..

[20] Masten A.S. (2014). Global perspectives on resilience in children and youth. Child Dev..

[21] Eysenck M.W., Derakshan N., Santos R., Calvo M.G. (2007). Anxiety and cognitive performance: Attentional control theory. Emotion.

[22] Hobfoll S.E., Halbesleben J., Neveu J.-P., Westman M. (2018). Conservation of resources in the organizational context: The reality of resources and their consequences. Annu. Rev. Organ. Psychol. Organ. Behav..

[23] Hayes A.F. (2013). Mediation, moderation, and conditional process analysis. Introduction to Mediation, Moderation, and Conditional Process Analysis: A Regression-Based Approach.

[24] Peng J., Liu Y., Wang X., Yi Z., Xu L., Zhang F. (2025). Physical and emotional abuse with internet addiction and anxiety as a mediator and physical activity as a moderator. Sci. Rep..

[25] Penninx B.W., Pine D.S., Holmes E.A., Reif A. (2021). Anxiety disorders. Lancet.

[26] Dai H.L., Yu Z.B., You L.Q., Fan M.H., Zhu H.W., Jiang D.J., Wu M.Y., Lin S.J., Zhang X.C., Chen K. (2019). Association between social health status and depressive symptoms among community-dwelling elderly adults in Zhejiang Province, China. J. Zhejiang Univ. Sci. B.

[27] Weinstein A., Maayan G., Weinstein Y. (2015). A study on the relationship between compulsive exercise, depression and anxiety. J. Behav. Addict..

[28] Khanzada F.J., Soomro N., Khan S.Z. (2015). Association of Physical Exercise on Anxiety and Depression Amongst Adults. J. Coll. Physicians Surg. Pak..

[29] Berggren N., Derakshan N. (2013). Attentional control deficits in trait anxiety: Why you see them and why you don’t. Biol. Psychol..

[30] Troy A.S., Willroth E.C., Shallcross A.J., Giuliani N.R., Gross J.J., Mauss I.B. (2023). Psychological Resilience: An Affect-Regulation Framework. Annu. Rev. Psychol..

[31] Fletcher D., Sarkar M. (2013). Psychological Resilience. Eur. Psychol..

[32] Neenan M. (2017). Developing Resilience: A Cognitive-Behavioural Approach.

[33] Polizzi C.P., Lynn S.J. (2021). Regulating emotionality to manage adversity: A systematic review of the relation between emotion regulation and psychological resilience. Cogn. Ther. Res..

[34] Bandura A. (2014). Self-efficacy mechanism in psychobiologic functioning. Self-Efficacy.

[35] Derakhshan N. (2020). Attentional control and cognitive biases as determinants of vulnerability and resilience in anxiety and depression. Cognitive Biases in Health and Psychiatric Disorders.

[36] Çakir G., Isik U., Kavalci İ. (2025). An evaluation of physical activity levels and mental health among young people: A cross-sectional study. BMC Psychol..

[37] Liu M., Liu H., Qin Z., Tao Y., Ye W., Liu R. (2024). Effects of physical activity on depression, anxiety, and stress in college students: The chain-based mediating role of psychological resilience and coping styles. Front. Psychol..

[38] Zhiyong L., Weitang L., Hadier S.G., Xiaoyuan Z., Hokun Y. (2026). A study on the correlation between knee muscle strength and agility in competitive Wushu Changquan athletes. Front. Physiol..

[39] Maxwell S.E., Cole D.A. (2007). Bias in cross-sectional analyses of longitudinal mediation. Psychol. Methods.

[40] Lwanga S.K., Lemeshow S. (1991). Sample Size Determination in Health Studies.

[41] Yang Y., Hadier S.G., Long L., Hamdani S., Hamdani S.D. (2025). Development and cross-validation of LMS-based normative reference standards and health benefits zones for muscular strength among adolescents by age and sex. Front. Public Health.

[42] Hamdani S., Zhuang J., Hadier S.G., Khurram H., Hamdani S.D.H., Danish S.S., Fatima S.U., Tian W. (2023). Establishment of health related physical fitness evaluation system for school adolescents aged 12-16 in Pakistan: A cross-sectional study. Front. Public Health.

[43] Fritz M.S., MacKinnon D.P. (2007). Required sample size to detect the mediated effect. Psychol. Sci..

[44] Masyn K.E. (2013). Latent class analysis and finite mixture modeling. The Oxford Handbook of Quantitative Methods in Psychology: Vol. 2: Statistical Analysis.

[45] Spitzer R.L., Kroenke K., Williams J.B., Löwe B. (2006). A brief measure for assessing generalized anxiety disorder: The GAD-7. Arch. Intern. Med..

[46] Ahmad S., Hussain S., Shah F.S., Akhtar F. (2017). Urdu translation and validation of GAD-7: A screening and rating tool for anxiety symptoms in primary health care. J. Pak. Med. Assoc..

[47] Campbell-Sills L., Stein M.B. (2007). Psychometric analysis and refinement of the Connor-davidson Resilience Scale (CD-RISC): Validation of a 10-item measure of resilience. J. Trauma. Stress.

[48] Mustafa G. (2016). Exploring Construct Validity of Resilience Scale in Pakistani Youth. J. Appl. Environ. Biol. Sci..

[49] Cho M.H. (2016). Preliminary reliability of the five item physical activity questionnaire. J. Phys. Ther. Sci..

[50] Ramaswamy V., DeSarbo W.S., Reibstein D.J., Robinson W.T. (1993). An empirical pooling approach for estimating marketing mix elasticities with PIMS data. Mark. Sci..

[51] Lo Y., Mendell N.R., Rubin D.B. (2001). Testing the number of components in a normal mixture. Biometrika.

[52] Nylund K.L., Asparouhov T., Muthén B.O. (2007). Deciding on the number of classes in latent class analysis and growth mixture modeling: A Monte Carlo simulation study. Struct. Equ. Model. A Multidiscip. J..

[53] Stults-Kolehmainen M.A., Sinha R. (2014). The effects of stress on physical activity and exercise. Sports Med..

[54] Schuch F.B., Stubbs B., Meyer J., Heissel A., Zech P., Vancampfort D., Rosenbaum S., Deenik J., Firth J., Ward P.B. (2019). Physical activity protects from incident anxiety: A meta-analysis of prospective cohort studies. Depress. Anxiety.

[55] Du X., Jiang F., Yuan S. (2025). A cross-sectional study on the correlation between exercise frequency and mental health among university students based on international physical activity questionnaire-short form classification: Evidence from undergraduate students aged 18–22. Front. Psychol..

[56] Qin T., Chen P., Wang J., Dong J., Zhang K. (2024). Impact of physical activity on anxiety among university students: A moderated mediation model. Front. Psychol..

[57] Eysenck M.W., Derakshan N. (2011). New perspectives in attentional control theory. Personal. Individ. Differ..

[58] Cameron L.D. (2012). Anxiety, cognition, and responses to health threats. The Self-Regulation of Health and Illness Behaviour.

[59] Wang W., Liu H. (2025). Canonical correlation analysis of anxiety symptom and behavioral inhibition/activation system among college students and their relationship with physical activity. Sci. Rep..

[60] Hadier S.G., Liu Y., Long L., Hamdani S.M.Z.H., Khurram H., Hamdani S.D., Danish S.S., Fatima S.U. (2024). Assessment of physical literacy in 8- to 12-year-old Pakistani school children: Reliability and cross-validation of the Canadian assessment of physical literacy-2 (CAPL-2) in South Punjab, Pakistan. BMC Public Health.

[61] Long L., Hamdani S.D., Hamdani S., Zhuang J., Khurram H., Hadier S.G. (2024). Establishing age- and sex-specific anthropometric growth references standards for South Punjab adolescents utilizing the LMS method: Findings from the Pakistani population. Front. Public Health.

[62] Hadier S.G., Yinghai L., Long L., Hamdani S.D., Hamdani S. (2024). Mediation role of cardiorespiratory fitness on association of physical activity and physical literacy among 8-12 years old children: The PAK-IPPL cross-sectional study. Front. Pediatr..

[63] Sharp P., Oliffe J.L., Kealy D., Rice S.M., Seidler Z.E., Ogrodniczuk J.S. (2023). Social support buffers young men’s resilient coping to psychological distress. Early Interv. Psychiatry.

[64] Sarkar M., Fletcher D. (2014). Psychological resilience in sport performers: A review of stressors and protective factors. J. Sports Sci..

[65] Karasz A., Gany F., Escobar J., Flores C., Prasad L., Inman A., Kalasapudi V., Kosi R., Murthy M., Leng J. (2019). Mental health and stress among South Asians. J. Immigr. Minor. Health.

[66] Basu D., Nagpal S., Mutiso V., Ndetei D.M., Lauwrens Z., Hadfield K., Singh S., Bhui K.S. (2020). Enhancing resilience and mental health of children and adolescents by integrated school-and family-based approaches, with a special focus on developing countries: A narrative review and call for action. World Soc. Psychiatry.

[67] Tomczyk S., Schomerus G., Stolzenburg S., Muehlan H., Schmidt S. (2018). Who is seeking whom? A person-centred approach to help-seeking in adults with currently untreated mental health problems via latent class analysis. Soc. Psychiatry Psychiatr. Epidemiol..

[68] Worsley J.D., Pennington A., Corcoran R. (2022). Supporting mental health and wellbeing of university and college students: A systematic review of review-level evidence of interventions. PLoS ONE.

[69] Meng Y., Wang Y., Liu Q., Liang C., Yang S. (2025). The relationship between physical activity, sleep, and negative emotions in physically weak college students. Front. Public Health.

[70] Jiang Y., Zhang B., Zhao H. (2025). Analysing the effect of physical exercise on social anxiety in college students using a chained mediation model. Sci. Rep..

[71] Javaid Z.K., Chen Z., Ramzan M. (2024). Assessing stress causing factors and language related challenges among first year students in higher institutions in Pakistan. Acta Psychol..

[72] Shah M., Hasan S., Malik S., Sreeramareddy C.T. (2010). Perceived stress, sources and severity of stress among medical undergraduates in a Pakistani medical school. BMC Med. Educ..

[73] Maqsood A., Gul S., Noureen N., Yaswi A. (2024). Dynamics of perceived stress, stress appraisal, and coping strategies in an evolving educational landscape. Behav. Sci..

