# Supplementary material for: Anxiety, Psychological Resilience, and Physical Activity Among University Students: Mediation and Latent Profile Analyses
Source: Healthcare (Basel). 2026 May 5;14(9):1246. doi: 10.3390/healthcare14091246 (PMC13163358; doi:10.3390/healthcare14091246)
Supplement: Supplementary file 1 [file healthcare-14-01246-s001.zip › healthcare-4176840-supplementary.pdf]

Questionnaire Code:

Health and Well-being Survey

This survey aims to understand your daily experiences and your ability to cope with life's challenges. Your answers are confidential and will be used for research/screening purposes.

Section A: Demographic Information

Instructions: Please check the box or write the answer that best describes you.

Name:	Contact:
Age (years):	Gender: [0] Male [1] Female [2] Other/Prefer not to say
City of Residence:	University:
Semester/Year of Study:	Major/ Subject Studying:
Marital Status<br>[0] Single/Never Married [1] Married<br>[2] Divorced/Separated [3] Widowed	Monthly Income (PKR/USD)<br>[0] No income ; [1] 6000–25000 ($38–$151) [2] 25000–40000 ($151–$242) [3] 40000–80000 ($242–$484) [4] 80000–170000 ($484–$1028)

Section B: Generalized Anxiety Disorder (GAD-7) Scale

Instructions: Over the last 2 weeks, how often have you been bothered by the following problems?

Over the last 2 weeks, how often have you been bothered by:	Not at all (0)	Several days (1)	More than half the days (2)	Nearly every day (3)
1. Feeling nervous, anxious, or on edge	0	1	2	3
2. Not being able to stop or control worrying	0	1	2	3
3. Worrying too much about different things	0	1	2	3
4. Trouble relaxing	0	1	2	3
5. Being so restless that it is hard to sit still	0	1	2	3
6. Becoming easily annoyed or irritable	0	1	2	3
7. Feeling afraid as if something awful might happen	0	1	2	3

GAD-7 total score: ________ / 21

Section C: Physical Activity Scale

Instructions: Please rate your habitual physical activity levels on a scale of 0 to 4.

**Please rate the following:**	**(0)**	**(1)**	**(2)**	**(3)**	**(4)**
**1. Frequency:** How often do you participate in physical activity in your free time?	Rarely or never	1–2 times per week	3 times per week	4–5 times per week	Every day
**2. Intensity:** How intensely do you participate in the activity?	Very light	Light	Moderate	Hard	Very hard
**3. Duration:** How many minutes do you typically spend per session?	< 20 mins	21–30 mins	31–40 mins	41–50 mins	> 51 mins
**4. Type:** What is the primary nature of your activity?	Sedentary	Light (stretching)	Moderate (jogging)	Vigorous (running)	High-impact
**5. Length:** How long have you maintained this level of activity?	< 1 month	1–3 months	4–6 months	7–12 months	> 1 year

Physical activity total score: ________ / 20

Section D: Resilience Scale (CD-RISC-10)

Instructions: Please indicate how much you agree with the following statements as they apply to you over the last month.

**Over the last month**	**Not at all true (0)**	**Rarely true (1)**	**Sometimes true (2)**	**Often true (3)**	**True nearly all the time (4)**
1. I am able to adapt when changes occur.	0	1	2	3	4
2. I can deal with whatever comes my way.	0	1	2	3	4
3. I try to see the humorous side of things.	0	1	2	3	4
4. Coping with stress can make me stronger.	0	1	2	3	4
5. I tend to bounce back after illness or hardship	0	1	2	3	4
6. I can achieve goals despite obstacles.	0	1	2	3	4
7. Under pressure, I stay focused and think clearly.	0	1	2	3	4
8. I am not easily discouraged by failure.	0	1	2	3	4
9. I think of myself as a strong person.	0	1	2	3	4
10. I can handle unpleasant/painful feelings.	0	1	2	3	4

CD-RISC-10 total score: ________ / 40
